# Robotic-assisted closed-chest management of a fungal-infected prosthetic aortic graft: a case report

**DOI:** 10.1186/s13256-022-03380-0

**Published:** 2022-05-10

**Authors:** Ashley T Giammarino, Iam Claire Sarmiento, SJacob Scheinerman, John Winalski, Richard S Lazzaro, Derek R Brinster, Jonathan M Hemli

**Affiliations:** 1 Touro College of Osteopathic Medicine, Middletown, NY USA; 2 Department of Cardiovascular and Thoracic Surgery, Lenox Hill Hospital/Northwell Health, 130 East 77th Street, 4th floor, New York, NY 10075 USA

**Keywords:** *Aspergillus*, Aortic graft, Robotic-assisted, Omentum, Case report

## Abstract

**Background:**

Fungal prosthetic graft infections are associated with high mortality, typically requiring aggressive surgical debridement. We present an alternative, minimally invasive approach to address these challenging clinical cases.

**Case presentation:**

A 76-year-old Caucasian male with prior aortic root and arch replacement presented with localized chest wall tenderness after being hit by a car door. Computed tomography angiogram incidentally identified fluid in the anterior mediastinum, surrounding his ascending aortic graft. Rather than undertaking a high-risk reoperative sternotomy and redo complex aortic reconstruction, we elected to proceed with a robotic-assisted, minimally invasive debridement of the aortic graft, coupled with an omental wrap, entirely within the closed chest. Microbiology was positive for *Aspergillus* species. The patient made an uncomplicated recovery and was discharged home on antifungal therapy, likely to continue indefinitely.

**Conclusions:**

Infected prosthetic aortic grafts can be successfully managed with debridement and pedicled omental flap coverage via a minimally invasive approach within the closed chest, obviating the morbidity of a complex reoperative open procedure.

## Background

Prosthetic vascular graft infections, although uncommon, are associated with high mortality [1]. Although traditional management typically involved graft excision and radical debridement, almost always via open approach [1, 2], the high morbidity inherent in these procedures has prompted a search for other potential treatment options, including extraanatomic bypass, graft irrigation, and use of autologous tissue grafts and/or xenografts [3]. Indeed, more recently, there has been a noticeable trend towards a more conservative approach in these challenging cases, given the mortality associated with graft explantation, whether it be coupled with inline or extraanatomic reconstruction [4].

The majority of prosthetic aortic graft infections tend to be bacterial, with Gram-positive organisms constituting at least half of all positive tissue cultures [5]. Fungal infections, although less common, have been reported, although typically in either single case reports or small case series, and have usually been described in immunocompromised patients or in those with other predisposing risk factors for fungemia, with *Candida* species being the most frequently identified pathogen [3, 6, 7]. Most patients with thoracic aortic graft infections tend to present with classic symptoms and signs indicative of sepsis, although these may be more subtle in the setting of less virulent organisms and include more nonspecific complaints, such as fatigue, malaise, and/or weight loss [8].

We present a case of fungal-infected aortic graft that was highly atypical in a number of ways, in that the patient was essentially asymptomatic and had no preexisting susceptibility to fungal contamination, the pathogen involved was an *Aspergillus* species (rather than a *Candida* organism), and, perhaps most importantly, the patient was successfully managed using a robotic-assisted, minimally invasive approach entirely within the closed chest, a surgical technique not commonly described for this highly morbid condition.

## Case presentation

A 76-year-old Caucasian male with history of acute Stanford type A aortic dissection, initially managed by aortic root and ascending aortic replacement, developed a pseudoaneurysm of his graft anastomoses one year thereafter, mandating reoperative aortic reconstruction and total arch replacement. Two years subsequent to this, he presented with localized chest wall pain after being struck by a car door. He demonstrated no overt symptoms of sepsis, was afebrile, and felt otherwise well. Clinical examination was largely unremarkable, notwithstanding localized chest wall tenderness corresponding to the point where he had sustained his recent trauma. More specifically, he exhibited no signs suggestive of endocarditis or even of any infective process in general. He had no leukocytosis or relative neutrophilia, although he did have moderately elevated biochemical inflammatory markers, specifically erythrocyte sedimentation rate (ESR) and C-reactive protein (CRP).

**Fig. 1.**
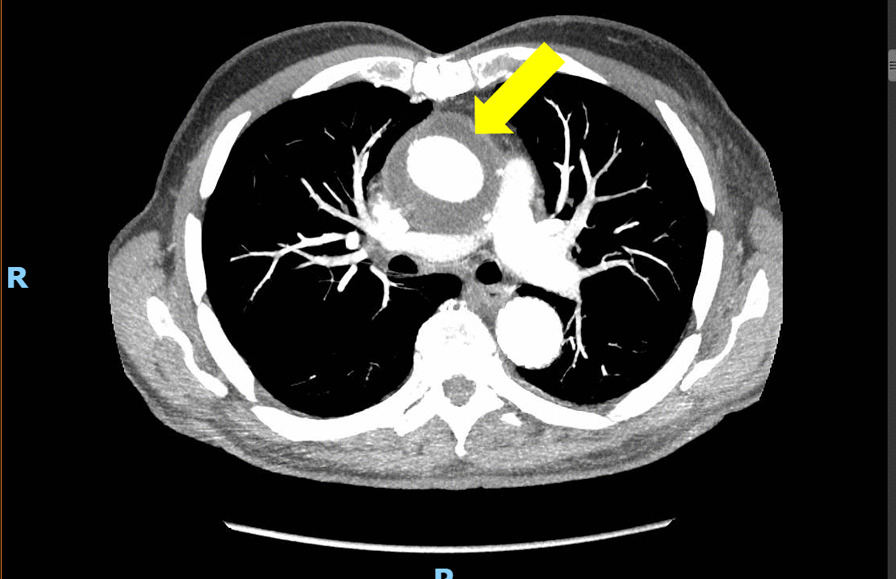
Computed tomography angiogram demonstrating significant fluid collection surrounding the prosthetic ascending aorta (arrow)

A computed tomography angiogram (CTA) incidentally demonstrated fluid surrounding his aortic graft, as well as in the left axilla, wherein he had had an extraanatomic bypass to his left subclavian artery as part of his reoperative total arch replacement (Fig. 1). An echocardiogram confirmed no vegetations on his bioprosthetic aortic valve.

The patient was taken to the operating suite for an initial diagnostic procedure. Purulent fluid was aspirated from the presternal area and from the left axilla. All sternal wires were removed, the presternal tissues were debrided, and a vacuum wound dressing was placed. The left axilla was opened and drained, and vacuum wound dressing was used at this site also. Microbiology was positive for *Aspergillus* species.

Given the inherent risks of another reoperative sternotomy with redo complex aortic reconstruction, a decision was made to approach further debridement in a nonconventional fashion, via a minimally invasive technique, with robotic assistance.

The patient was returned to the operating room for more definitive treatment. Utilizing the da Vinci robotic platform (Intuitive Surgical, Sunnyvale, CA), the prosthetic ascending aorta was exposed through the right chest, utilizing three ports. Aggressive debridement of the aortic root, ascending aorta, and proximal arch were undertaken utilizing the robotic instruments.

Part of the greater omentum was then harvested, using typical laparoscopic techniques, but with the assistance of the robot. The vascularized pedicled omental flap was then passed cephalad through a defect created in the diaphragm, just to the right lateral aspect of the central tendon. This was wrapped around the prosthetic ascending aortic graft and tacked into position with a number of interrupted polypropylene sutures.

**Fig. 2.**
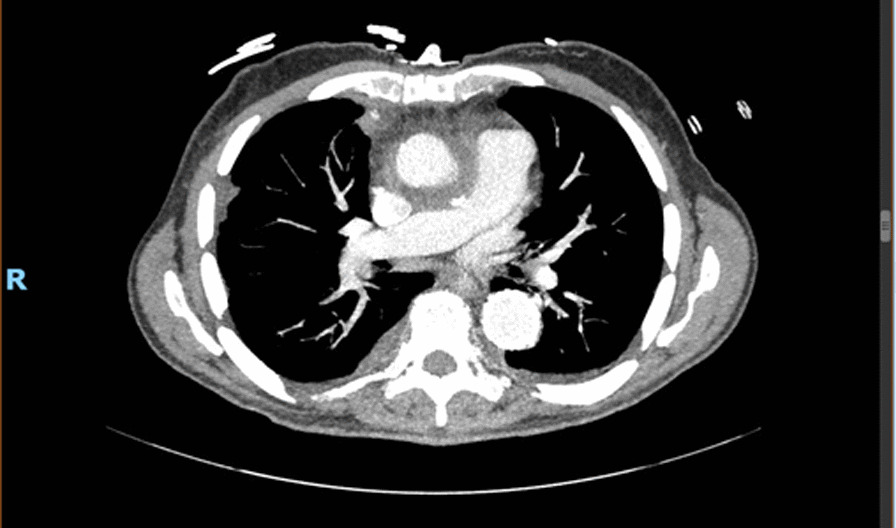
Computed tomography angiogram obtained 1 week postoperatively, demonstrating substantially decreased fluid around the ascending aorta

**Fig. 3.**
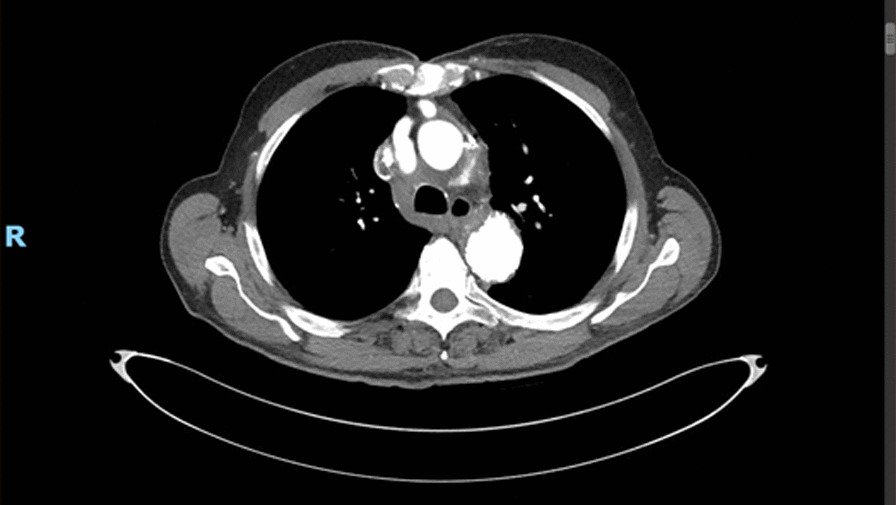
Computed tomography angiogram at 18 months’ follow-up, essentially demonstrating no residual perigraft collection

The patient made an uncomplicated postoperative recovery. After 48 hours in the intensive care unit, he was transferred to a regular telemetry bed, where he continued to demonstrate good progress. A repeat CTA obtained one week postoperatively demonstrated significantly less periaortic fluid (Fig. 2). The patient was then discharged home on indefinite antifungal therapy, as advised by Infectious Diseases, given that we had left all of his prosthetic graft material *in situ*. At 18 months’ follow-up, the patient remained clinically well, and interval CTA (Fig. 3) confirmed no reaccumulation of perigraft fluid.

## Discussion

Current surgical dogma for an infected prosthesis suggests aggressive debridement with removal and replacement of the graft [9]. Although this may have traditionally constituted the classic gold standard of care, we deemed a further reoperative sternotomy and aortic reconstruction to be of prohibitive risk for our patient under discussion, given his two prior aortic procedures, which had already included repair of anastomotic dehiscence and total arch replacement [10]. The Society of Thoracic Surgeons online risk calculator (https://riskcalc.sts.org/stswebriskcalc/calculate) does not specifically take into consideration procedures on the thoracic aorta; as a corollary, however, our patient’s calculated risk of surgical in-hospital mortality was 33.95% by Euroscore II (https://qxmd.com/calculate/calculator_285/euroscore-ii). We, however, believe that this calculated risk actually still markedly underestimates his true operative hazard, given that the Euroscore II algorithm incorporates a field only for “thoracic aorta surgery” in general, without specifying the type of operation that would be undertaken. In our case, to remove all infected and contaminated graft, we not only would we have been forced to undertake a third-time sternotomy and redo aortic root replacement, but we would have been mandated to replace the entire aortic arch for a second time, including bypasses to all of the great vessels, as well as redo reconstruction of the extraanatomic graft to the left upper extremity. Given this prohibitive surgical risk, and in light of the fact that *in situ* graft-sparing surgery has been increasingly reported as safe and effective, for both early and late prosthetic graft infection, and that it has been associated with mortality benefits over more radical techniques of graft explantation and reconstruction [11, 12], we sought to explore alternative treatment strategies.

It is interesting to note that our patient did not clinically present with typical features suggestive of sepsis. Lyons and associates suggested guidelines to assist in establishing the diagnosis of prosthetic graft infection, based on clinical, radiological, and laboratory findings [13]. Recent literature suggests that most patients with an infected aortic graft tend to present with at least some clinical evidence of sepsis [14], but this was not the case in our patient, in which the periaortic collection was an incidental radiologic finding after he was hit in the chest wall by a car door. Our patient’s fluid cultures yielded *Aspergillus*, another unusual finding because the most common infectious agents in these cases, as previously alluded to, are Gram-positive bacteria, typically *Staphylococcus aureus* [15].

Harky and colleagues summarized numerous approaches to the management of proximal aortic graft infections; all the proposed surgical strategies involved an open technique [14]. Given the increased risk of mortality associated with redo sternotomy, these investigators acknowledged antibiotic therapy as being an acceptable starting point for therapy, but they ultimately concluded that graft removal was the only feasible definitive treatment option. Even if we had elected to undertake a third-time aortic procedure in our patient, a cadaveric cryopreserved aortic homograft alone would have been insufficient to replace the entirety of his prosthetic aortic root, ascending aorta, and aortic arch, such that we would have still been mandated to implant new prosthetic material into an infected surgical field. By deciding to leave the entirety of his prosthetic aorta in place, using a minimally invasive, robotic-assisted technique to debride the infection and then fill the perigraft space with vascularized tissue, our case thus challenged these more traditional principles.

The advantages of using greater omentum in sternal wound and thoracic graft infections have been well described [16]. The omentum provides a diverse network of cells that induce a strong immune response, which not only helps to combat infection but also supports tissue growth and wound healing [17]. In this way, the omentum is not dissimilar to secondary lymphoid tissue, with an innate ability to induce a strong immunogenic response via cytokines and other agents, including vascular endothelial growth factor (VEGF), transforming growth factor (TGF)-β, and platelet-derived growth factor (PDGF), all of which stimulate angiogenesis and collagen synthesis [18]. A vascularized omental flap also offers the advantages of being easily harvested, pliable, and able to be molded and wrapped around the ascending aorta in its entirety, eliminating as much of the contaminated dead space as possible, while delivering antibiotics to the tissues, maintaining adequate lymphatic drainage, and maximizing graft–flap contact [19]. Our patient had the advantage of not only avoiding a further reoperative sternotomy, but also of having his omentum harvested laparoscopically, with robotic assistance, which has been shown to reduce the incidence of secondary surgical-site infection, as well as promote faster recovery time and improve postoperative pain [20, 21]. Although intercostal muscle, pectoralis major, and rectus abdominus flaps can all be used to deliver vascularized tissue to an infected aortic graft, these options all have their demerits when compared with the use of pedicled omentum, including relative inability to reach deeply within the chest, lack of pliability to more completely cover the aortic graft and thoroughly obliterate any dead space, and possibly not inconsiderable donor-site morbidity [22, 23].

There are only a limited number of cases to date that have reported using a thoracoscopic approach to debride an infected aortic graft entirely within the closed chest [24]. Additionally, to the best of the authors’ knowledge, this case report is one of the first to describe employing robotic-assisted techniques, not only to debride the infected aortic graft, but also to harvest and place the omentum as a periaortic wrap. Although others have used a laparoscopic approach to harvest omentum, a sternotomy was almost invariably performed in these cases to place and cover the aortic graft [15, 25].

## Conclusion

We suggest that the minimally invasive technique described herein, achieving all of our management goals entirely within the closed chest, holds promise for those patients deemed at prohibitive risk for redo complex aortic surgery, and that surgeons should consider this option in these challenging cases.

## Data Availability

At the request of the reader.

## Abbreviations

CRP: C-reactive protein
CTA: Computed tomography angiogram
ESR: Erythrocyte sedimentation rate
VEGF: Vascular endothelial growth factor
TGF: Transforming growth factor
PDGF: Platelet-derived growth factor
